# The regulation and reconciliation of hybrid professional-managerial identities in a public hospital: the case of Lean management

**DOI:** 10.1108/JHOM-02-2024-0058

**Published:** 2025-05-08

**Authors:** Adamina Ivcovici, Greg Bamber, Timothy Bartram, Pauline Stanton, Jessica Borg, Patricia Pariona-Cabrera

**Affiliations:** Yarra Health Services, Richmond, Australia; Monash University, Melbourne, Australia; RMIT University, Melbourne, Australia

**Keywords:** Hybrid professional managers, Hospital, Identities, Improvement, Lean

## Abstract

**Purpose:**

This study aims to examine how hybrid medical managers in a public hospital reconcile their identities and involvement in management-introduced top-down interventions to improve operational performance. In our study, Lean serves as an example of a management intervention through which we examine how hybrids shape the implementation of managerial interventions in a public hospital.

**Design/methodology/approach:**

We gathered our data from a longitudinal qualitative study of a Lean initiative implemented in an Australian public hospital. The current analysis is part of a larger case study which involved 87 in-depth semi-structured interviews over three years in a major Australian public hospital. These interviews explored experiences of Lean and included senior managers, middle managers, hybrids, clinical staff and others. In this paper, we focus specifically on the experiences of hybrid medical managers.

**Findings:**

We demonstrate how the Lean initiative sparks identity-reconciliation work that differs among hybrids working in different parts of the hospital and with various contractual arrangements and levels of participatory voice. The hybrids in our study responded to the introduction of Lean, with heightened identity reconciliation work, but in different ways. This appears to be attributable to the organisational context, and particularly the hybrids’ contractual arrangements with the hospital.

**Originality/value:**

There is a dearth of research in healthcare management that has sought to understand how hybrids reconstruct their identities in response to top-down implementations of managerial initiatives, such as Lean. Our findings offer healthcare managers practical insights into the engagement of hybrid-medical professionals through our novel understanding of the identity-reconciliation work necessary for hybrid professionals to engage with management initiatives.

## Introduction

In recent years, hybrid medical managers (hybrids) have grown in importance within healthcare organisations (Kippist and Fitzgerald, 2009). The term refers to the combined clinical and managerial roles undertaken by doctors in a healthcare setting (Braithwaite and Hindle, 2001). In this way, the duality of the role comes with its own set of challenges, notwithstanding the complexity of an individual needing to alternate and balance the tasks pertaining to both managerial and clinical objectives (Kippist and Fitzgerald, 2009). Individuals in these hybrid roles play a pivotal part in the management of healthcare organisations. They are critical players who engage in organisational improvement efforts. This is particularly true in public healthcare organisations where hybrid medical managers can wield significant influence by virtue of their clinical professional identities and associated status. It is important for scholars to unpack how and why hybrids engage with the implementation of management interventions (often conceived by senior management and functional managers) which are intended to reduce costs and improve efficiency and healthcare service delivery.

One such management intervention introduced in the healthcare sector is Lean Management (Lean). Lean management was developed from the Toyota production system and initially revolutionised manufacturing (White *et al*., 2013) by its focus on continuous improvement and reducing non-value-adding processes (Drotz and Poksinska, 2014). The concept of Lean has since been introduced in many healthcare contexts in attempts to contain costs and improve service quality and performance. Despite this, in the healthcare context, Lean practices have not become the “panacea” that some had hoped (Radnor and Boaden, 2008). Simultaneously, hybrids have grown rapidly (Burgess and Currie, 2013; Noordegraaf, 2015). Given that hybrids play important boundary-spanning roles in mobilising practices such as Lean into professionalised clinical settings, it is important to explore the role of hybrids while considering the context of management interventions being implemented. In this way, Lean as a management intervention provides us with an example through which we can explore the way medical hybrids shape the implementation of managerial interventions in hospitals. Examining the identity construction of hybrid medical managers is important given that management innovations are often imposed by management, driven by cost savings and efficiency gains (Bartram *et al*., 2020). Understanding how medical hybrids can more effectively engage with the development and implementation of management interventions is important for the sustainability of healthcare organisations and doctors exercising their professional and ethical responsibilities. Moreover, understanding the movement between professional and managerial identities is important for both scholars and practitioners because it provides new insights into the processes through which medical hybrids engage with change management to influence and adapt management interventions, to both protect the interests of the doctors and the quality of patient care (Cooke and Bartram, 2015).

Furthermore, while some continue to advocate the potential of hybrids to mobilise efficiency and quality agendas in healthcare, this potential has not been realised as easily as some managers and policymakers had hoped (Kislov *et al*., 2016; Sartirana *et al*., 2019). This mixed success may be due to hybrids having to face challenges negotiating the tensions between medical and managerial logics (Byrkjeflot and Kragh Jespersen, 2014; Llewellyn, 2001; Schott *et al*., 2016). This can be attributed to their complex and often politicised role as both managers and medical professionals (Denis *et al*., 2015; Llewellyn, 2001; Waring, 2014).

There has been much research to explore the challenges of hybrids at the micro-level (e.g. Croft *et al*., 2015; Fitzgerald and Ferlie, 2000; Kitchener, 2000; Llewellyn, 2001; McGivern *et al*., 2015; Sartirana *et al*., 2019; Spyridonidis *et al*., 2015). However, relatively little research in healthcare management has sought to understand how hybrids reconstruct their identities in response to the top-down implementation of managerial initiatives such as Lean, particularly when such interventions are introduced because of policy-level efficiency pressures. Nevertheless, such research is important as the successful implementation of Lean in public health requires the engagement and commitment of clinicians (Leggat *et al*., 2015; Stanton *et al*., 2014).

Moreover, as Fitzgerald and Ferlie (2000) argue, analyses of management efficiency reforms have tended to present them as unilateral macro-level “challenges” to the traditional logic of professionalism. The relationship between hybrids and managerial interventions which aim to address policy-level efficiency pressures has been neglected in the literature. To address this neglect, we examine the practices through which hybrids negotiate and shape the implementation of Lean as a managerial intervention in a public hospital, as well as the implications of their practices both for their hybrid identities and for the management intervention. To help us achieve this, we use two theoretical key concepts. Firstly, we draw on the concept of “identity reconciliation” (Wenger, 1998) which underlines that we exist in a “nexus of multi-membership” in various communities of practice with which we identify more or less strongly and that our various identities must constantly be reconciled to maintain a sense of coherence. Secondly, we deploy Alvesson and Willmott’s (2002) theoretical concept of “identity-regulation” which considers the effects of organisational practices on processes of identity construction.

Combining these theoretical lenses, we conceptualise identity reconciliation as *a process of reconciling one’s identity with one’s engagement with managerial interventions (in our case, using Lean as an example of a management intervention)* and recognise the potential of such interventions to regulate and shape identities. This lens enables us to focus on the *practices* through which hybrids engage with management interventions rather than the *outcomes* of interventions like Lean a problem in much of the literature about Lean management. This is critical to understand questions about *how* and *why* initiatives like Lean are negotiated and shaped by actors as they try to reconcile their identities with their engagement in such interventions.

Bartram *et al*. (2020) examined how hybrids contributed to organisational change during the introduction of Lean from an institutional theory perspective. In this article, we dig deeper to understand the identity reconciliation work underpinning their ability to engage with Lean in the first instance and to successfully enact practice changes during Lean implementation.

Our research questions are:

1. How and why do hybrids reconcile their identities through their engagement with managerial interventions?
2. How do hybrids’ identity reconciliation practices shape managerial interventions?

To address these questions, we examine how hybrids in the emergency department (ED) and surgical department of a major public hospital undertook identity reconciliation in response to the initiative of Lean management. We gathered our data from a longitudinal qualitative study of a Lean initiative implemented in an Australian public hospital.

We make two main contributions to understanding how and why healthcare hybrids influence the practice of managerial interventions. Firstly, using the theoretical lenses of identity reconciliation and regulation, we show how hybrids reconcile their identities in response to the introduction of management interventions, using Lean as an example. Secondly, we draw attention to the link between hybrid identity reconciliation processes and the practice of managerial interventions in public hospitals, and how each is recursively shaped by the other. We build on Bartram *et al*. (2020) to reveal how different contractual arrangements and sub-organisational contexts impact engagement with and commitment to Lean implementation.

We organise the article as follows. Firstly, we provide a review of the literature on hybrids. Secondly, we introduce the theoretical concepts of identity reconciliation and identity regulation and show how this combined lens enables us to consider the implementation of Lean as an identity-regulating mechanism. Thirdly, we outline our study, introducing the top-down introduction of Lean at the hospital where we conducted our research, and summarise our research methods. Fourthly, we present our findings using narrative fragments to illustrate perspectives on hybrid engagement with Lean. To conclude, we discuss the theoretical and practical implications of our research for healthcare management.

## Literature review

This section provides a review of the literature on the key concepts in relation to hybrid-medical professionals. In this way, the following sections will provide the theoretical background upon which our study is built. Firstly, we discuss lean healthcare as an improvement approach via three key principles with a focus on reducing non-value-adding processes. Secondly, we provide theoretical background on hybrids, focused on public healthcare contexts at a macro level, as well as different responses of medical professionals. Thirdly, we appraise the extant literature on hybrids, including the duality of doctors as managers and healthcare professionals. We consider the role of doctors, acknowledging that their skills, knowledge and experience contribute to the effectiveness of the healthcare system. Finally, we provide an explanation pertaining to the transition of medical professionals to a hybrid role, looking closely at the impacts at a micro level, including internal tensions, transitions and hybrid construction.

### Lean healthcare

Lean in healthcare can be used as a quality improvement methodology to reduce costs and improve healthcare service delivery (Moraros *et al*., 2016; Stanton *et al*., 2014). Lean is an influential management approach with its origins dating back to early US post-war process improvement and contributions by quality specialists (i.e. Deming and Juran). Lean emphasises standardisation, process improvement and elimination of excess inventory (Sohal *et al*., 2022). There are three main principles of Lean: standard work promotion; ongoing accountability and oversight; and connection between leadership and frontline workers to drive daily continuous improvement (Winner *et al*., 2022; Rathi *et al*., 2022). This study uses Lean as an example of a management intervention and interaction with existing clinical practice.

LM is often attractive to organisations facing significant challenges in difficult healthcare contexts (Bartram *et al*., 2020). Waring and Bishop (2010:1334) argue that LM “illustrates the desire of policy makers to reorder clinical work through the introduction of managerial philosophies and techniques”. There are mixed findings regarding the success of LM initiatives in healthcare contexts. Some studies have linked Lean with improved process outcomes in healthcare settings (Mahmoud *et al*., 2021; Kovacevic *et al*., 2016), as well as enhancements in labour productivity and quality of care (Tiapa; Poksinska *et al*., 2017). For example, Stanton *et al*. (2014) reported that LM initiatives had a positive impact on the quality of work life of nurses and doctors and their level of participation and control over their work. In contrast, other studies have reported that Lean in healthcare is challenging to implement and does not always lead to improvements in efficiency or healthcare outcomes. Alternatively, authors like McCann *et al*. (2015:1557) have rejected “the current prescriptive or managerial discourses on lean” in healthcare. His study of a large UK health service reported that despite LM initially being seen as a beacon of “hope”, it was later cancelled (McCann *et al*., 2015). Moreover, Leggat *et al*. (2015) argued that barriers such as lack of a culture of continuous quality improvement, skilled leaders, inadequate resources and poor implementation of Human Resource Management (HRM) may contribute to the lack of success of Lean.

### Hybrids in public healthcare

At macro levels, research has shown how professional work in public service organisations has been reconfigured by macro-level policy changes, for instance, through the standardisation of work practices and performance measurement (Evetts, 2009; Kirkpatrick *et al*., 2015). In public healthcare contexts, scholars have argued that reforms and their effects have been shaped by the responses of medical professionals. For example, doctors have responded to changes through professional re-stratification, adapting existing systems to reflect their own policy aspirations and circumventing management systems by emphasising their superiority (Currie *et al*., 2012; Waring and Currie, 2009).

Such macro changes can be seen at the organisational level in the evolution of hybrid roles, such as clinical directorships. Structurally, medical hybrids hold positions in between top levels of (usually non-clinical) management and (clinical) frontline managers. They often maintain their clinical practice and therefore their professional legitimacy, while also contributing to the implementation of organisational policies and practices (Sartirana *et al*., 2019). There is much interest in legitimising hybrid positions based on their potential to improve collaboration between managers and doctors and improving the development and implementation of management initiatives. This is evident through the establishment of medical management as a medical profession subspecialty. However, there is reluctance among medical professionals to take up hybrid roles (Ferlie, 2016).

### Medical manager duality

Doctors are instrumental in the healthcare sector and their capacity to employ medical and management skills are critical and affect change at all levels (Grady and Hinings, 2019). Research has confirmed that the role of doctors as stewards of change is critical in which their management, leadership, engagement and accountability for outcomes are important to the provision of healthcare (Bergevin *et al*., 2016). As doctors, they focus on patient care, and they are well-placed to explain patients” challenges. Doctors in a management capacity can influence change that is grounded in clinical practice (Grady and Hinings, 2019). When doctors as primary service providers are equipped and engaged with the right mix of skills, which are rarely acquired during medical training (Blumenthal *et al*., 2012), they can play a pivotal role as leaders in healthcare organisations (Fournier and Jobin, 2018). According to Denis *et al*. (2013, p. 22), this is viewed as “conversion to an asset for the [healthcare] system when physician expertise, legitimacy as professional and substantial influence is capitalized upon”.

Regardless of the recognised value of the important role of doctors as both clinicians and managers in the healthcare system, challenges remain (Fournier *et al*., 2022; Kaissi, 2005; Sartirana and Giacomelli, 2024). It is difficult to balance doctors’ individual interests (as autonomous professionals) with the collective interests of health services. Doctors as managers/leaders must continually demonstrate how both roles are related and resolve possible tensions between their individual and collective interests (Grady and Hinings, 2019). Empson (2017) stated “[doctors] are generally reluctant to see themselves as followers and may be equally reluctant to put themselves forward as leaders” (p. 4). Similarly, Bohmer (2012, p. 26) noted that “frontline doctors are unprepared and unschooled for a management/leadership role, often unsupported in this work”. However, research conducted by Moller and Kuntz (2013) showed that the effects of managerial responsibilities on the orientation as a professional are critical. They found that hospitals benefit from doctors who have “entrepreneurial and managerial skills, as well as professional skills” (p. 15). Importantly, professional orientation should not be in conflict with managerial responsibilities. Therefore, the dual role of doctors is important for healthcare services (Diamond, 2004; DeRue and Ashford, 2010). Doctors can manage patients’ day-to-day tasks and they can understand what is happening at the “floor level” (Domecq *et al*., 2014; Grady and Hinings, 2019). They can also influence the broader need for improvement in healthcare organisations by using their knowledge of patient care deliver at the “balcony level” (Fisher *et al*., 2009; George *et al*., 2013; Goodall, 2011). Therefore, as Denis *et al*., (2023, p. 3) highlighted “a focus on healthcare system improvement probably suggests a new modus operandi between the system, the organisation and the profession”.

### Transition to a hybrid role

At micro levels, the reluctance of doctors to become medical hybrids is reflected in studies of individual hybrid identities. These demonstrate the challenges hybrids face, predominantly with regard to reconciling tensions between professional and managerial principles (e.g. Croft *et al*., 2015; Kitchener, 2000; Llewellyn, 2001; Spyridonidis *et al*., 2015). The potential of hybrids to act as a “bridge” between management and medical professionals is dependent upon complex processes of identity construction.

Firstly, professionals face internal tensions between the often different logics and values of professionalism and managerialism and perceptions from the frontline “rank-and-file” that clinical managers have gone over to “the dark side” can exacerbate this tension (Croft *et al*., 2015). Currie *et al*. (2009b) demonstrate the particular challenges of hybridisation when it is associated with enacting or supporting government policy.

Secondly, “transitions” are challenging. Hybrids’ capacities to influence across the two groups rely on their ability to construct a positive liminal space and to use “two-way windows”, which allow them to move between professional and managerial identities (Croft *et al*., 2015; Llewellyn, 2001). Spyridonidis *et al*. (2015) demonstrate how new “nested” identities can be achieved if clinicians begin from the position of a secure and salient cross-cutting identity of “being a clinician” (as experienced by senior physicians) because there is a relatively low threat to such secure identities. These hybrids more readily align managerial tasks with their clinical identity and engage with organisational improvement practices. However, certain clinicians (usually junior ones) tend to perceive managerial roles as threatening their status and identity as competent clinicians. McGivern *et al*. (2015) contrast “incidental hybrids” who protect traditional professionalism with “willing hybrids” who are able to reconstruct their identity to integrate professionalism and managerialism, facilitated by formative experiences and role models.

Thirdly, hybrid identity construction is not only dependent on the resolution of internal conflicts between individual and intra-professional challenges but also on social interactions with other groups in the wider organisational context. Sartirana *et al*. (2019) show that identity work is distributed and enabled by social interactions with actors beyond their professional group.

The research outlined above shows us that the process of *becoming* a hybrid requires not only reconciling internal tensions but also that hybrids are aware of and attempt to manage the perceptions of those they influence through their “two-way windows”. This process is distributed across social interactions in the broader organisational context and is influenced by macro-institutional contexts. There remains a need, however, to connect such micro-level understandings of hybrid identity with broader contexts including meso-organisational and macro-policy levels (Swan *et al*., 2016). Little explicit attention has been paid to the link between hybrid identity reconciliation processes and the practice of managerial interventions which are introduced to address policy-level efficiency pressures in hospitals, and how each is shaped by the other. Moreover, analyses of hybrids’ agency are scarce but are vital if we are understand their potential to impact the practice of management innovations and implications for doctors’ workloads and quality of patient care (Byrkjeflot and Kragh Jespersen, 2014; Numerato *et al*., 2012; Waring and Bishop, 2010). As Pratt *et al*. (2006) argue, there is a need to examine how identity and work reinforce each another.

Table 1 summarises the key points of our literature review relating to hybrid-medical professionals (see Table 2).

**Table 1:** Literature review summary table

Main elements in the literature	Brief description
Lean healthcare	The premise of Lean principles in healthcare is to reduce costs and improve service delivery
	Lean includes three main principles: standardised work processes; ongoing accountability and oversight; and connection between leadership and frontline workers to drive daily continuous improvement
	Research highlights both the benefits as well as the challenges of adopting Lean principles in healthcare
Hybrids in public healthcare	Medical-hybrids can have non-clinical and clinical roles and hold positions of top, middle and frontline managers. In their role, medical-hybrids perform their medical practice/duty whilst also contributing to the implementation of organisational policies and practices. Research highlights reluctance among medical professionals to take up hybrid roles with doctors reluctant to be viewed as followers and/or leaders
Medical-manager duality	Doctors play an important role in the quality of patient care and they can leverage this “on-the-ground’ knowledge in their managerial responsibilities in the healthcare system. When able to balance their day-to-day client-facing role with a managerial role, doctors can influence change that is grounded in clinical practice
The transition to a hybrid role Internal tension Transitions Hybrid identity construction	At a micro-level, research highlights the factors that make the transition of doctors and medical professionals into a hybrid role difficult. The medical-hybrid role involves individuals who operate as both clinicians and managers. Research shows the following factors as impacting individuals in such roles Different logics, values and perceptions are present among hybrid clinicians Having a two-way role as both professional and manager leads to challenges Individual and intra-professional challenges, as well as social interactions are present

**Source(s):** Authors’ work

**Table 2:** Interviews used in this study

Type of participant	Total interviews	Participant code
Non-clinical managers	3	CEO
		Non-clinical manager 1
		Non-clinical manager 2
Hybrid nurse	1	Senior nurse manager
Hybrid doctor-managers	5	Medical Director, ED
		Deputy Director, ED
		VMO (Lead Surgical Consultant)
		Medical Department Director 1
		Medical Department Director 2

**Source(s):** Authors’ work

## Theoretical framework

### Making the connection between hybrid identities and the practice of management interventions: identity reconciliation and regulation

To enhance understanding of the interplay between hybrids’ contextually situated identity work and management interventions such as Lean, we draw on Wenger’s notion of “*identity as reconciliation”* (1998, p. 159) and Alvesson and Willmott’s (2002) concept of *identity regulation*.

In Wenger’s view, we become who we are through our practices, which are socially situated. We always participate in multiple “communities of practice” (e.g. medical, managerial, non-work communities), and identity is an experience of this “nexus of multi-membership” (Wenger, 1998). This ensures that identity is precarious – a dynamic “uncertain truce” (Byrkjeflot and Kragh Jespersen, 2014). To maintain a coherent self-identity across boundaries and time, some reconciliation of these different identities is necessary (Wenger, 1998).

Despite its significance and complexity, identity reconciliation happens privately, is never complete and may not be consciously acknowledged by the individual. Consequently, the influence of individual participants’ identity reconciliation on managerial interventions is rarely considered in practice or in research. Moreover, Wenger (1998) does not provide guidance as to how to theorise what is involved in identity reconciliation.

Handley *et al*. (2006) prefer Alvesson and Willmott’s notion of identity regulation to explain identity reconciliation and to understand the motivations for actors embracing or rejecting opportunities to participate in organisational activities. Identity regulation encompasses the effects (intentional or not) of social practices (e.g. rewards, organisational structures, training and promotion) on processes of identity construction. The concept thus invites an appreciation of the interplay between managerial identity-regulating mechanisms, and other elements of hybrids’ identities. Hence we consider a management intervention (in our case, Lean) to be an identity regulating mechanism in the sense that organisational control “*is accomplished through the self-positioning of employees within managerially inspired discourses about work and organisation with which they may become more or less identified and committed”* (Alvesson and Willmott, 2002, p. 620).

We therefore understand *identity reconciliation* as a negotiation between organisational discourses that potentially regulate and shape hybrids’ identities (intentionally or not) and other sources of identity in their nexus of multi-membership to establish a sense of continuity and coherence over time and across situations (Alvesson and Willmott, 2002, p. 625). This conceptualisation allows us to locate hybrids’ identity reconciliation within the specific practice of their hybrid work where these negotiations take place (e.g. when implementing Lean). Also, because actors negotiate their identity through their practices, this lens also enables us to see how hybrids negotiate and shape the practice of Lean through the identity reconciliation process (Wenger, 1998).

In deploying the combined theoretical lenses of identity reconciliation (Wenger, 1998) and identity regulation (Alvesson and Willmott, 2002), we see that the introduction of an intervention such as Lean represents a situation in which heightened identity reconciliation work is likely to be required of hybrids given that it may have implications doctors’ work processes, workloads and quality of patient care (Korica and Molloy, 2010; McGivern *et al*., 2015). We use this lens to elucidate the practices involved in hybrids’ identity reconciliation work in response to managerial interventions and explore how such interventions can be reconfigured following this reconciliation work.

## Research context: lean as a management intervention through which to explore hybrids’ role in reconciling identities

Perceived poor performance of EDs, in terms of efficiency and access targets, is a political issue internationally. Consequently, governments have established performance targets, such as the “four-hour rule” in Australia. This is adapted from the UK National Health Service (NHS) Eight-Hour Rule, a performance indicator requiring that 80% of patients arriving in EDs were admitted or discharged within 8 h (Stanton *et al*., 2014). In response to such policy pressures to improve efficiency, as well as budgetary constraints, managers of hospitals have increasingly implemented Lean practices since the 1990s.

In our study, Lean serves as an example of a management intervention through which we can explore how hybrids shape the implementation of managerial interventions in public hospitals. In our case, we researched the implementation of Lean in a major Australian public hospital. The Chief Executive Officer (CEO) of this hospital initiated the Lean project after several years of poor ED performance, which had attracted unwanted attention from policymakers and the media. The Lean projects focused on achieving better hospital-wide patient flow to address issues of overcrowding and long stays in the ED and were implemented in parallel across various departments. As we show later, Lean was implemented in a top-down way involving little consultation and some difficult interactions between executive managers and key hybrids.

## Research approach, data collection and analysis

This analysis is part of a larger case study which involved 87 in-depth semi-structured interviews, which were collected over three years (2012–2015) in a major Australian public hospital. The hospital was a large tertiary facility in an urban setting with approximately 800 beds, over 10,000 staff and an operating budget of over $1 billion. The operations of the ED within the hospital were comparable to other Australian hospitals. The hospital was chosen as it met our selection criteria of being a large, tertiary, urban hospital that was also an early adopter of Lean practices. These interviews explored experiences of Lean practices and included senior managers, middle managers, hybrids, clinical staff and others. Interview participants were obtained with the help of the human resource department at the hospital, through which emails were sent out inviting interested individuals who met our selection criteria based on role and title, to participate. We also sought to obtain further participants through snowballing.

The data for this article are drawn from analyses of nine of these interviews (see Table 1) – three with managers, two key hybrid doctor-managers connected to emergency departments (employed by the hospital), two senior hybrid doctor–managers from other departments (employed by the hospital) and a visiting medical officer (VMO) hybrid (the lead surgical consultant who is not employed by the hospital). We limit our focus to a few key players to generate deep “experience-near” (Froggett and Briggs, 2012; Geertz, 1974) insights into the identity reconciliation practices of a small number of actors. This “*small n”* approach facilitates abductive theory refinement rather than deductive theory testing and helps us to achieve a “*clearer view”* of identity reconciliation and regulation (Tsoukas, 2019). Semi-structured interview guides were developed. These were guided by the research questions, as well as the healthcare literature. There was also an element of co-design with the hospital, whereby the challenges of the hospital were factored into our research questions to ensure that the participants could reflect on the Lean practices within the hospital setting they were familiar with.

With permission of the interviewees, we audio-recorded interviews. We analysed these transcripts using inductive thematic content analysis to try to identify meanings in our qualitative data (Patton, 2015). Three independent coders analysed the transcripts. We coded transcripts manually, using NVivo software to store data and easily search for keywords and content. Firstly, we applied descriptive codes to the data. These were derived “bottom-up” from our interview transcripts and were also sensitised by the constructs we brought to the data from the literature, and our knowledge of the context. This process led to the key finding that there were significant differences between hybrids’ engagement with Lean in different organisational subcontexts. Secondly, we compared and contrasted between the ED hybrids and the VMO. Thirdly, we considered relationships between the emergent findings, applying an abductive logic (Yin, 2014) to build explanations about how hybrids’ identity reconciliation influences management interventions.

We wove our data into narratives which elaborate the aspects of identity reconciliation and regulation that emerged. We followed narrative approaches to represent and understand change in the healthcare management field (Currie *et al*., 2009a; Currie and Brown, 2003; Mcdonald *et al*., 2005; McDonald *et al*., 2006). Such approaches have not to our knowledge been used earlier to focus on hybrids. This seemed to be an effective approach through which to build and counterpose the differing perspectives of the non-clinical managers, ED hybrids and VMO hybrids.

## Findings

### Lean initiative

The hospital in our study implemented a Lean management improvement initiative with the aim of facilitating greater efficiency in patient flows through the accident and emergency (A&E) department. This aimed to reduce the time for patients to be seen and attended to within the A&E department, with an objective to meet targets of 8-h maximum wait times. The CEO of the hospital was a key player in driving the Lean initiative which was referred to as “the 8-h project”. To facilitate the Lean initiative, the hospitals received additional resources from the government. The Lean management approach involved hospital staff being trained by an external team from a commercial enterprise, as well as support by the hospital Quality Improvement Team.

### Implementing lean from the top-down

The CEO of our hospital decided to implement Lean as a management intervention. She saw it as a way to turn around poor financial performance and achieve the four-hour rule. However, there was early resistance from hybrids:

… [clinical directors] said that [Lean] wouldn’t work in a clinical setting … That the clinical staff … wouldn’t participate in the project … they couldn’t see that Lean … had any meaning whatsoever for a hospital. (CEO)

From the beginning, the CEO framed hybrids as resistant and incapable of seeing the benefits of Lean that managers could see, even though hybrids and their clinical staff had not been engaged in selecting Lean (management intervention).

An early interaction between the CEO and a senior medical director was mentioned by multiple interviewees. It demonstrates the “us” and “them” dynamic between two key groups that emerged (or re-emerged) at one of the early Lean meetings. This senior hybrid suggested that they might take time to reflect on what had been learnt through successive quality improvement attempts over many years. In response, the CEO drew attention to her position at the top of the hierarchy. The senior hybrid stated:

I put up my hand and said, “Listen, I’ve been through this twice. Why don’t we get the executive summaries of [earlier] reports and table them … that information will be valuable in directing this new project.” Do you know what [CEO] said to me? “Would you sit down, that’s not relevant, that’s old data.’ I said, “ … whenever I write a scientific paper, I have to do a literature review, and in the introduction, you acknowledge previous publications to set the scene for this new project. I disagree with you.” And I was belittled in front of 30 nurses, who were on the committee. Thirty! (medical department director 1)

This senior hybrid worked to publicly identify herself not only as a medical professional by drawing attention to specific knowledge and skills that distinguished her from the CEO (by implication, lacking scientific research skills) but also as having expertise in quality improvement, drawing attention to her hybridity. In response, the CEO publicly enacted her dominant identity. The interaction signified the CEO’s jurisdictional claim over organisational improvement, her hierarchical location, and her attempts to establish “rules of the game” in relation to the Lean implementation (Alvesson and Willmott, 2002, p. 631).

Opportunities to develop specific Lean knowledge and skills through training were offered inconsistently to medical clinicians and hybrids, who were under-represented on Lean projects compared with other clinical groups. Non-clinical managers cited doctors’ lack of time as a reason for this and also suggested the training was too basic for them.

… they don’t need to sit in a classroom to understand this stuff. You give [them] a book and they’d read it and they’d know it … these are not foolish people. (non-clinical manager 1)

Whereas the CEO had earlier identified doctors as unable to understand the benefits of Lean, the above suggests a different kind of regulatory influence on hybrids’ identities. The excerpt above suggests that non-clinical managers controlled and constrained the opportunities hybrids and clinicians had to gain Lean knowledge and skills by making direct appeals to their medical identity as high-status, intelligent professionals.

At other times, non-clinical managers strategically sought hybrids’ involvement, deciding whose voices were heard:

… the decision to get her [onto the Lean project] was around me … getting her a voice with the CEO … we gave [medical hybrid] … the conduit, or the pathway … to get a voice …. (non-clinical manager 2)

The implication is that managers were necessary advocate for hybrids and doctors, who otherwise had little voice in relation to the implementation of the Lean initiative. As in the earlier examples, this manager reinforces the notion that doctors are important, but simultaneously constructs a medical professional identity of a diminished role where managers, who control Lean resources and expertise, are required to empower and enable hybrids and doctors to act on their improvement ideas.

Further evidence of managers controlling medical involvement is seen in the way managers related with those who did not seek to be involved.

I … selected some of the more difficult characters … and pulled them into the project. Because if you get [them] on side, you move mountains. There was a couple of the medical [people] who were without a doubt belligerent and difficult … very cynical … so I … said, “Let’s have a conversation about this.” Because … they have an enormous influential power out there that needs to be working with us, not against us … (non-clinical manager 1)

As the quote demonstrates, hybrids are influential and powerful, but the non-clinical managers are identified as leaders of the Lean work who not only enable involvement by providing a “conduit” but also strategically “pull” certain hybrids in, perhaps against their wishes. As a senior nurse manager put it, doctors were inclined to “*cooperate … But they weren’t actively a part of it”.* The common thread here is how non-clinical managers channelled hybrids’ involvement with the Lean projects by identifying themselves as the dominant players in the implementation of the management initiative.

These narrative fragments show how the discourses and actions of non-clinical managers, including the CEO, had identity-regulating implications for hybrids. There was top-down control regulation of training opportunities for hybrids – variously justified as “protecting” their time (as hybrids were highly valued, had high status and were “above” learning about Lean) or as “enabling” them to have a voice on Lean projects (implying hybrids were less expert, less empowered members of the Lean “community of practice”).

Next, we turn to the responses of senior hybrids in the ED and later compare these to the response of the VMO hybrid in the surgical unit. We show how their situated identity reconciliation resulted in Lean being practised differently, as they negotiated the Lean implementation in relation to their differing contractual arrangements with the hospital and their different suborganisational contexts.

### ED hybrids – soft regulation and smooth reconciliation

In terms of hospital-wide patient flow, the ED was the most obvious example of poor performance. There were reports of “excessive” wait times, overcrowding and long lengths of stay in the ED, which attracted attention from the media, public and by policymakers monitoring hospital performance. This put pressure on ED staff. The ED team experienced more criticism than the rest of the hospital. The key hybrids in the ED were longstanding hospital employees, and, as a hybrid from another area of the hospital described, ED doctors are highly committed team players. In ED, there was a sense that, “*it doesnt actually matter if the rest of the world is against you, as long as you’ve got the team behind you” (medical department director 2)*.

While medical staff rarely undertook Lean management training, the ED was an exception. Recognising the impact Lean could have on the ED’s work, the ED medical director, enacting the managerial aspect of her hybrid identity, introduced a departmental key performance indicator (KPI) requiring all medical staff in the ED to complete a minimum level of Lean training.

I introduced [the training KPI]. I thought, “We’re going to have this language, we need to all understand the language.” So, I’ve asked all of my Emergency physicians to spend two days learning it, so that they – we can all participate. (medical director ED)

The ED director believed that by ensuring her team received Lean training, her department would be better placed to relate with managers, and she worked to identify her group as *insiders* to Lean practice. The ED team was thus better able to negotiate the practice of Lean and retain some control over the changes.

Interviews with other doctors in the ED implied they felt that the pressures on the ED were poorly understood by other doctors, resulting in their being unfairly blamed for issues related to patient flow and wait times. Lean had the potential to pinpoint the cause of these issues – beyond the ED. This had motivated the deputy director to *“[have] time committed to this”* and, like the ED director, she attended weekly Lean meetings with the executive. Apart from recognising the influence that identifying with and belonging to the Lean “in-group” would afford the department, she felt that engaging with Lean would engender empathy across the hospital and that it was “*fantastic for breaking down the silos”.*

Becoming allied with managers was a way to bring to the ED power and influence on the Lean projects and within the hospital more generally. However, there was more to hybrid involvement than simply obtaining influence by being part of the Lean “in-group”. The hybrid leaders of the ED identified with managers’ efforts to enhance the understanding between disparate medical departments and came to view Lean as a rigorous methodology.

You can’t just say, … we’ve got an idea of what to do, let’s work out how to do it.’ But I think by getting everyone involved in stepping through each part of it and reviewing it, it helps reinforce the idea that this is being done rigorously. (deputy director ED)

While Lean (as the management intervention) and managers faced resistance due to the perception of “foreign” values and rationality among many doctors, this was not the case in ED. Lean provided a forum for ED doctors to share their department’s issues. The success of the ED projects was, in a sense, non-negotiable as KPIs were imposed by policymakers and bonuses were allocated to executives for their achievement. While ED doctors may not have received Lean training had their medical director not pushed it, there was a place reserved for them at the weekly Lean meetings with the CEO. With a voice in the decision-making in relation to the Lean initiative and perceiving shared interests, relational ties and affiliation between these doctors and managers were reinforced. This conveyed a sense of solidarity and shared identity and made it easy for these hybrids to reconcile their clinical and managerial selves through the practice of Lean. Consequently, in this organisational sub-context where ED hybrids were long-term employees of the hospital, who saw themselves as integral to the functioning of the overall hospital, the implementation of Lean faced little resistance and ED hybrids were committed participants in the implementation process.

### VMO – distance and resistance

The lead surgical consultant, a hybrid VMO, constructed a very different engagement with Lean and resisted reconciling his identity in a way that would see him “cave in” to attempt to regulate his professional identity and practice. VMOs provide their services to the hospital, including within the ED, on a part-time basis. In contrast to the ED doctors above who were full-time employees, these clinicians were essentially independent contractors and had therefore always been “outsiders” with regard to policy and process.

The truth is … surgeons just come in and do work … …. And if we decide that something ain’t right, we have to work out ways *de novo* of trying to fix it. (Lead surgical consultant – VMO)

The VMO said “*my position doesn’t actually come with any power”,* and before the implementation of Lean, he did not see himself or his peers as part of the broader medical community at the hospital: “*There aren’t specific … forums where we would specifically interact [with other clinicians]”.*

When Lean was introduced, the VMOs’ outsider identities were reinforced by their exclusion from consultation about changes in the ED where they provided surgical services. Despite this, this hybrid explained that they also appreciated the potential of Lean as an opportunity to access resources to achieve their own existing improvement agenda, which they had previously sought executive support for without success. The innovation involved a change in the model of medical assessment that would reduce time to diagnosis and treatment, thereby improving patient flow.

Led by the hybrid ED director, a group of VMOs banded together to garner support for their idea. Despite occupying the hierarchically advantageous position of head of the surgical department, our hybrid VMO eventually concluded that because of their lack of voice as hospital “outsiders” due to their contractual arrangements, the surgeons’ improvement ideas would be heard by non-clinical managers only if they promoted their ideas aggressively through the lens of Lean.

[The CEO] instituted [Lean]. We weren’t involved with that from the start …. We [VMO surgeons] only got involved because we annoyed everybody a lot … seeing that this [four-hour rule] thing was happening, we thought we’ll do a three-week pilot without any funding … We were working outside the system … being as annoying as we possibly could and trying to use names that stuck in people’s minds … that actually got the hospital executive on board. Because previously they had been uninterested in this process.

It was many months into implementation that “*suddenly we had a voice at the table”*. This hybrid admitted, however, that he was hardly a genuine participant – “*really I was evilly trying to use the process for the end that I wanted”*. As another medical hybrid from the hospital reiterated, Lean had been seen by many doctors as “*some bureaucratic imposition” (medical department director 2).*

The VMOs appeared to not only accept their outsider identity but to further it by constructing themselves as a rival tribe to the non-clinical managers who were directing the Lean projects. This hybrid and his team were defiant in the face of their exclusion and used aggressive labels for their team and projects, calling themselves *“guerilla improvers”* to signal distance between “us” (medical professionals) and “them” (non-clinical managers and their bureaucratic imposition).

Interestingly, it was through this approach that the VMOs were able to obtain the resources and support they had long sought, but unsuccessfully. The VMO’s narrative reveals that capturing the attention of managers to influence the Lean improvement work was a means to achieving the improvements he felt were important for his department’s patients. They engaged only eventually, through necessity, and on their terms. As VMOs working as independent contractors, they had never felt “part of” the hospital before and did not expect or necessarily want this to change. However, the non-clinical managers had given the VMOs a voice at the table.

## Discussion

In addressing our research questions, we sought to understand how and why hybrids reconcile their identities through their engagement with managerial interventions, and how their identity reconciliation practices shape such interventions. The hybrids in our study responded to the initiative of Lean as an introduced management intervention, with heightened identity reconciliation work, but in different ways. This appears to be attributable, to some extent, to the organisational context, and particularly their contractual arrangements with the hospital. This allows us to build on earlier work (Bartram *et al*., 2020; Waring and Currie, 2009), by examining the role that different organisational contexts play in the identity reconciliation work of hybrids.

There were similarities in terms of identity reconciliation and the practice of Lean across the ED hybrids and the VMO. Both hybrid types had pre-existing agendas that were specific to their organisational context. Both reconciled their existing hybrid identity, in which quality improvement (e.g. the managerial discourse of Lean) was valued within an identity oriented towards delivering the best possible patient care. Moreover, both were able to construct Lean as a valuable discourse to incorporate in their hybrid “toolkit” as a way to improve the operation of ED and quality of patient care. They viewed Lean as a way to build relationships and influence with non-clinical managers, and as a strategy to maintain autonomy and control over the seemingly inevitable practice changes that would result from Lean – with or without their engagement. However, the rationale, level of affiliation with non-clinical managers and level of autonomy they sought, differed. This had significant implications for their identity reconciliation practices.

ED hybrids were able to reconcile their existing identities with the newly introduced discourses of Lean. The approach to implementing Lean in the ED might be considered a “soft” form of identity regulation. The implementation had a regulatory effect through its stimulation of identity reconciliation, but reconciling the practice of Lean with the ED hybrids’ identities came with significant instrumental benefits (e.g. may have improved their perceived ability to provide efficient and high-quality patient care) and was therefore a relatively “smooth” process. Where medical hybrids regarded the Lean intervention as having merit to systematically improve the operation of the ED, they were more likely to engage with the development and implementation of Lean, as it was consistent with their medical values and interests.

We also sought to explore how hybrids’ identity reconciliation practices shape managerial interventions. Our findings illustrate that through engaging with Lean, and affiliating with managers as Allies, the identity of “team-working emergency doctor” could be reconciled with the Lean ideal of a whole-of-hospital team. In this way, by incorporating Lean practices into their existing hybrid repertoires, the hybrids’ existing agendas to break down professional silos (e.g. managers versus doctors) could also be realised. As in McGivern *et al*.’s (2015) study, our “willing” ED hybrids were able to integrate professionalism and managerialism in a productive way to improve the operation of the ED with potential implications for doctors’ workloads and quality of patient care. The identity-regulating effect of Lean was tangible – “*I thought, we’re going to have this language, we need to all understand the language”,* but bolstering the “managerial side” of their identity and that of ED frontline clinicians (in a way, hybridising the frontline) also strengthened the “professional side”. Identity reconciliation was productive in the sense that it brought resources to professionals in the form of managerial support, but also gave them control over how Lean was developed and implemented in the ED. Building relations with managers and internalising Lean was a way for doctors to maintain autonomy – an identity that allowed them to negotiate how Lean was implemented in their intra-organisational space.

On the other hand, the identity regulating influence of Lean discourses and practices on the VMO hybrid appeared less “potent” (Alvesson and Willmott, 2002). Lean was less readily reconciled with this particular hybrid’s existing identity. It appeared that the saliency of the VMO hybrid’s sub-community identity as a hospital “outsider” (and that of his “guerrilla” colleagues) was significant (Spyridonidis *et al*., 2015). This resulted in further distancing from the hospital, an adversarial stance and resistance to managers’ attempts to control the implementation of Lean and exclude the VMOs from the process. This situation – being outside formal structures, processes and procedures, and with less legitimate claim to hospital resources due to their “outsider” status was further enacted through the VMO hybrid’s engagement with Lean. This categorisation was further strengthened through his articulation of difference and distancing. He and his colleagues engaged with Lean, but in their own way. They defended their traditional discourses of professional dominance, and the VMO hybrid countered the potential identity-regulating effects of Lean by engaging with and enacting the discourse, but only insofar as it would help him to achieve his agenda (Alvesson and Willmott, 2002). He achieved this in ways that were defensive, to preserve the identity stability that comes with a cross-cutting professional identity (Spyridonidis *et al*., 2015). As a result, we suggest that senior VMOs may not see themselves as hybrids and that their hybridity may only become salient when their existing professional interests clash with their managerial responsibilities, and their identity requires negotiation and reconciliation. Further, we suggest that the distance created between the organisation and the VMOs by their contractual arrangements plays a particularly significant role not only in the hybrid professionals’ identity reconciliation in relation to Lean or other managerial interventions but also in how non-clinical managers relate to these relative “outsiders”. We summarise key findings and observations from our study in Table 3.

**Table 3:** Key findings and observations from our study

Main variables in our study	Key findings and observations
1. Lean management implementation	*Although the lean initiative was driven by the CEO and management, there was resistance from medical clinicians and hybrids towards lean implementation
	*There was an observed “us” versus “them” dynamic, whereby hybrids and clinical staff were framed by management as resistant to the Lean management intervention prior to the commencement of the initiative
	*Issues around training sessions (e.g. inconsistency, lack of time and quality) were observed by medical clinicians and hybrids
	*Non-clinical managers controlled and hindered the opportunities of hybrids pertaining to Lean training, etc.
2. ED hybrids’ positive approach	*Negotiations in relation to contractual arrangements and sub-organisational context contributed to an enhanced lean implementation process.
	*The ED served as an organisational sub-context in which ED hybrids were long-term employees of the hospital, who saw themselves as integral to the functioning of the overall hospital. In this case, the implementation of Lean faced little resistance and ED hybrids were committed participants in the implementation process and included in regular Lean meetings
2.1 ED-Identity reconciliation	*Identity reconciliation practices contributed to proactive lean implementation process via quality improvement and quality of patient care
2.2 ED-Identity regulation	*Soft form of identity regulation contributed to ED implementation of lean due to regulatory effect
3. Hybrid visiting medical officer (VMO)	*Hybrid VMOs experienced lack of voice related to the implementation of lean management. This can be attributed to their status as “outsiders” due to their “visiting” occupation
	*Hybrid VMOs were distanced from the hospital and excluded from the implementation of lean management

**Source(s):** Authors’ work

## Implications for healthcare management theory

We make two main theoretical contributions to understanding how and why hybrid medical professionals reconcile their identities in response to managerial interventions, and how these responses influence the practice of managerial interventions. Firstly, using the combined theoretical lenses of identity reconciliation (Wenger, 1998) and identity regulation (Alvesson and Willmott, 2002), we have elucidated identity reconciliation work, which remains under-researched in the healthcare context. In doing so, we show that hybrids’ identity reconciliation is heightened in response to the introduction of management interventions (i.e. Lean), which have certain (though highly variable depending on context and interpretation) identity-regulating effects. Critical to hybrids’ engagement with change managers and the intervention is the extent to which they regard it as beneficial to them and their patients. Moreover, an important part of identity regulation and reconciliation of hybrids is the role of change managers positively engaging with hybrids and encouraging their involvement in the development and implementation of interventions.

This has allowed us, firstly, to build on the study by Bartram *et al*. (2020) to show how contractual arrangements in different organisational contexts impact engagement with and commitment to Lean development and implementation. Secondly, we have drawn attention to how identity-reconciliation processes and the practice of managerial innovations in a public hospital setting are recursively shaped by each other. These contributions provide healthcare management scholars with a more nuanced understanding of how and why hybrid professionals influence top-down managerial attempts to improve healthcare services. Importantly, the need to improve efficiency, reduce wait times and enhance the quality of patient care, may motivate the identity-reconciliation work of medical hybrids.

By highlighting the importance of existing identities and different intra-organisational subcontexts and contractual arrangements in attempts to implement improvements in the quality of patient care in a healthcare setting, we elucidate the motivators and barriers of identity reconciliation work of hybrids. In this way, we provide fresh insights into why hybrids get involved in management interventions. This lens highlights the importance of recognising the potential identity-regulating effect of their practices, and of taking into consideration existing hybrid identities and the inevitability of identity reconciliation work in response to managerial interventions. Providing genuine opportunities for participatory decision-making and voice into improvement interventions, particularly for “contractually distant” hybrids, is important.

### Implications for healthcare management practice and future research

Our study has practical implications. Firstly, our findings suggest the importance of accounting for different actors’ identity-reconciliation practices in relation to management interventions. Hybrids must constantly reconcile their “nexus of multi-membership” within various communities of practice (e.g. as their profession, subspeciality, management role and organisational context), and in particular in response to organisational mechanisms of identity regulation (Alvesson and Willmott, 2002). Hybrids must navigate the development of top-down interventions and the expectations/goals of the quality management team which has implications for their identity regulation, in that they must negotiate and influence the intervention, and its effect on their staff and the quality of patient care. Medical hybrids may need to operate in a capacity as *protector* of their functional area and staff from potentially adverse effects and unintended consequences of top-down interventions (Bartram *et al*., 2020). The duality of the role necessitates that medical hybrids manage both the expectations of change managers, as well as those of their staff members and professional medical colleagues.

Secondly, ensuring that hybrids, especially those who may be contractually “distant” to the organisation, have a participatory voice into changes, is important for genuine engagement with the organisational implementation of managerial interventions. This may help inform the designers and implementors of management interventions to take into consideration the voices of disparate contractually engaged medical hybrids. For example, some participants in our study felt excluded from the managerial decision-making process (especially those who were contractually distant) concerning the management intervention (e.g. Lean). Our research suggests that by including hybrids in the decision-making process of new management interventions, hybrids can have input into the design of interventions, ensuring they consider their needs and those of the teams they manage. In this way, managerial and quality management teams may be able to better assist hybrids in reconciling their complex identities as both medical professionals and managers.

### Limitations of our research

There are of course limitations to our study. Given that our data examines the case of one public hospital in an Australian context, our results may not be generalisable to other healthcare organisations or contexts. We acknowledge the limitation of case study research as not being able to offer generalisability of our findings (Yin, 2018). Importantly, the aim of our research was not to make such statistical generalisations but rather to shed light on the under-researched phenomenon of identity reconciliation and construction in medical hybrids in response to managerial interventions. In taking a “*small n”* approach, we did not seek to produce statistical generalisability but rather “*analytical refinement”* and “*a clearer view”* (Tsoukas, 2019: p. 386) of identity reconciliation and regulation.

Moreover, we used Lean as an example of a management intervention to help us shed light on answering our research questions concerning hybrid identity construction and reconciliation. While this helped us advance research concerning the construction of hybrid identities with respect to management interventions, we cannot generalise that hybrid identity construction is the same for all management interventions. We therefore recommend that future research conduct similar studies using management interventions beyond Lean. These will help identify whether identity construction for hybrids differs based on different interventions or whether the process of identity construction is more static.

Furthermore, our conclusions may offer broad insights into a range of other settings, and we propose that future research explores whether this is the case. In particular, the context for public health in Australia is broadly comparable with other national contexts, especially in other developed market economies such as New Zealand, the UK and Canada, where public hospitals are funded primarily by taxation. Moreover, we assert that our insights regarding the relationship between identity reconciliation and regulation are valuable for management practitioners in other professionalised contexts in the public sector.

## Conclusion

We have responded to calls for explanations of variations in practice that take into account a range of contextual factors (Fitzgerald and Ferlie, 2000) and explore in detail the implementation of Lean as a management intervention and interaction with existing clinical practices (Waring and Bishop, 2010). We have shown that different organisational contexts (which may result from varying governance or contractual arrangements, pay structures and work organisation) open different frames for identity reconciliation in response to managerial interventions with identity-regulating effects. This is likely to influence the extent of “genuine” engagement with management interventions and make headway towards a better practical understanding and reconciliation of hybrid identities.
